# The Thermodynamic Stability of Membrane Proteins in Micelles and Lipid Bilayers Investigated with the Ferrichrom Receptor FhuA

**DOI:** 10.1007/s00232-022-00238-w

**Published:** 2022-05-13

**Authors:** Cosmin L. Pocanschi, Jörg H. Kleinschmidt

**Affiliations:** 1 Fachbereich Biologie, Universität Konstanz, 78457 Constance, Germany; 2 Institut Für Biologie, FB 10 Mathematik Und Naturwissenschaften, Universität Kassel Heinrich, Plett-Str. 40, D-34132 Kassel, Germany

**Keywords:** Thermodynamic stability, Outer membrane protein, Membrane protein folding, Lipid bilayer, Detergent micelle, *β*-barrel

## Abstract

Extraction of integral membrane proteins into detergents for structural and functional studies often leads to a strong loss in protein stability. The impact of the lipid bilayer on the thermodynamic stability of an integral membrane protein in comparison to its solubilized form in detergent was examined and compared for FhuA from *Escherichia coli* and for a mutant, FhuAΔ5-160, lacking the N-terminal cork domain. Urea-induced unfolding was monitored by fluorescence spectroscopy to determine the effective free energies $\Delta G{^\text{o}_{\rm u}}$ of unfolding. To obtain enthalpic and entropic contributions of unfolding of FhuA, $\Delta G{^\text{o}_{\rm u}}$ ﻿were determined at various temperatures. When solubilized in LDAO detergent, wt-FhuA and FhuAΔ5-160 unfolded in a single step. The 155-residue cork domain stabilized wt-FhuA by $\Delta\Delta G{^\text{o}_{\rm u}}$﻿~ 40 kJ/mol. Reconstituted into lipid bilayers, wt-FhuA unfolded in two steps, while FhuAΔ5-160 unfolded in a single step, indicating an uncoupled unfolding of the cork domain. For FhuAΔ5-160 at 35 °C, $\Delta G{^\text{o}_{\rm u}}$ increased from ~ 5 kJ/mol in LDAO micelles to about ~ 20 kJ/mol in lipid bilayers, while the temperature of unfolding increased from *T*_M_ ~ 49 °C in LDAO micelles to *T*_M_ ~ 75 °C in lipid bilayers. Enthalpies $\Delta H{_{\rm M}^\text{o}}$﻿were much larger than free energies $\Delta G{^\text{o}_{\rm u}}$﻿, for FhuAΔ5-160 and for wt-FhuA, and compensated by a large gain of entropy upon unfolding. The gain in conformational entropy is expected to be similar for unfolding of FhuA from micelles or bilayers. The strongly increased *T*_M_ and $\Delta H{_{\rm M}^\text{o}}$ observed for the lipid bilayer-reconstituted FhuA in comparison to the LDAO-solubilized forms, therefore, very likely arise from a much-increased solvation entropy of FhuA in bilayers.

**Graphical abstract:**

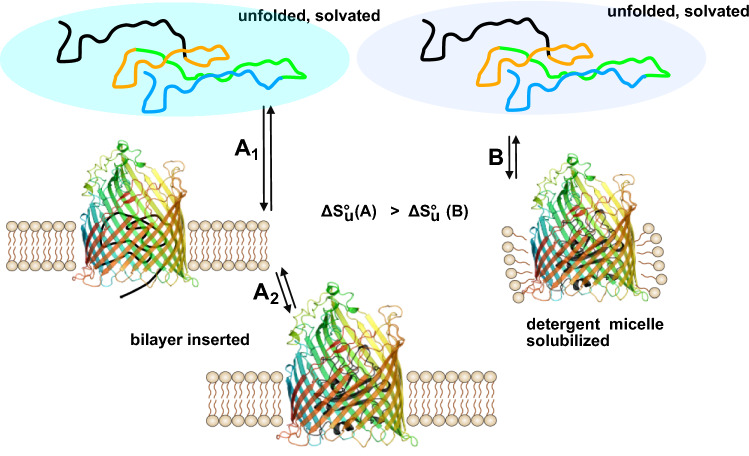

## Introduction

For structural and functional studies, transmembrane proteins (TMPs) are frequently extracted and isolated into detergent micelles. This solubilization often leads to partial inactivation/inhibition of TMPs, see e.g., refs. (Champeil et al., 2016; Miles et al., 2011; Zhou et al., 2001). Detergent molecules equilibrate between complexes, in which they associate with the TMP to cover its hydrophobic surface, detergent monomers in solution and detergent micelles free of TMP. The exchange rates of the detergent molecules between the states of the detergent molecules are rapid (Garavito & Ferguson-Miller, 2001; le Maire et al., 2000), and this frequent exchange at the interface of the membrane protein with the micelle is likely to contribute to the destabilization of integral membrane proteins in detergent micelles, which is one of the reasons why researchers are investigating alternatives to the classical detergent-micelle solubilization of membrane proteins *cf.* (Chaudier et al., 2002; Marty et al., 2013; Orwick-Rydmark et al., 2012; Stangl et al., 2014; Tribet et al., 1996).

Experimental examinations of the loss of the thermodynamic stability of a TMP caused by solubilization in detergent micelles are relatively rare. Differences in the melting temperatures *T*_M_ of TMPs in detergent-micelle vs. lipid membrane environments obtained by heat-induced denaturation have been determined for the *α*-helical Na/K-ATPase isolated from shark, with *T*_M_ ~ 10 °C lower in C_12_E_8_ detergent than in native membrane preparations of this ATPase (Miles et al., 2011). Experimental comparisons of the free energies of unfolding, $\Delta G{^\text{o}_{\rm u}}$﻿, of a selected TMP from micelle and bilayer environments have not yet been reported. Heat-induced unfolding of detergent solubilized TMPs often leads to their aggregation and precipitation and, therefore, complicates determinations of $\Delta G{^\text{o}_{\rm u}}$.﻿ *α*-helical TMPs are very hydrophobic and equilibria between folded states in membranes and unfolded forms are difficult to obtain. In contrast, *β*-barrel TMPs are composed of amphipathic *β*-sheets and, therefore, have a much lower average hydrophobicity than *α*-helical TMPs. Reversible folding and unfolding conditions have been reported for several *β*-barrel membrane proteins that become soluble at higher concentrations of chaotropic denaturants like guanidinium chloride or urea. Klug et al. (Klug et al., 1995) reported free energies of unfolding of the 22-stranded *β*-barrel ferric enterobactin receptor (FepA) from Triton-X-100 micelles. Hong and Tamm established conditions and reported free energies for the reversible folding of the 8-stranded *β*-barrel outer membrane protein A (OmpA) in phospholipid bilayers of small unilamellar vesicles (SUVs) (Hong & Tamm, 2004). As FepA and OmpA are very different in size and structure, the impact of the differences between the two environments detergent micelles vs. lipid bilayers on their different stabilities is not clear.

**Fig. 1:**
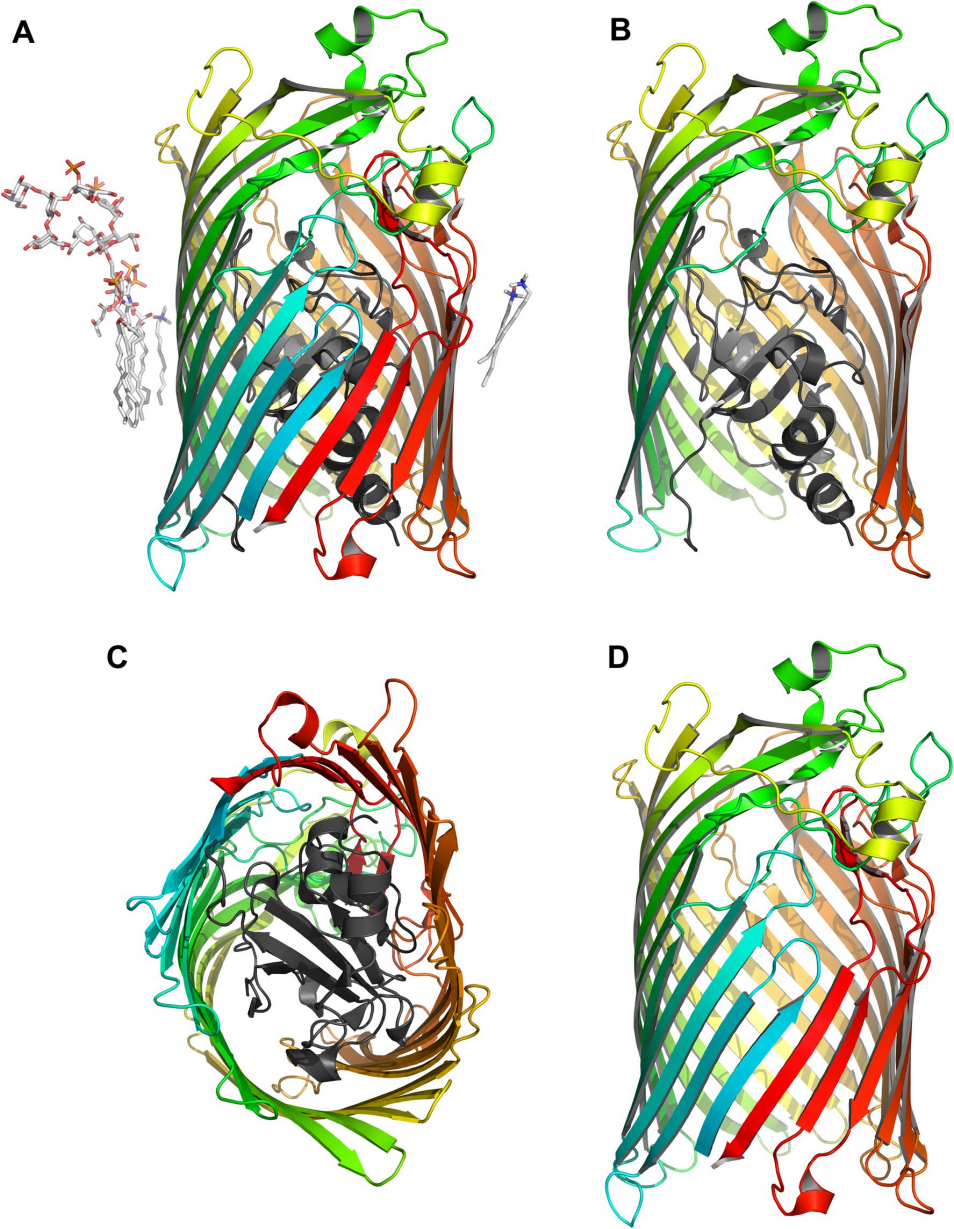
Crystal structures of FhuA (1QFG) (Ferguson et al., 2000). The cork domain is shown in black and the *β*-barrel domain in rainbow colors. **A** wt-FhuA in complex with one lipopolysaccharide (LPS) (left) and three dimethyldecylamine-*N*-oxide (DDAO) molecules (one on the left of FhuA and two on the right of FhuA). **B** wt-FhuA with the *β*-strands of the front of the *β*-barrel removed. **C** periplasmic view of wt-FhuA showing the location of the cork domain inside the *β*-barrel domain. **D** *β*-barrel domain of FhuA after removal of the cork domain

To examine and compare the thermodynamic stabilities of a *β*-barrel TMP in lipid membranes vs. detergent micelles, we selected the active iron transporter FhuA (Coulton et al., 1983; Fecker & Braun, 1983) from the outer membrane (OM) of *Escherischia coli*. FhuA (78 kDa, 714 residues) is a two domain TMP, comprised an N-terminal 160-residue domain termed “cork domain” located inside a 554-residue transmembrane domain that forms a 22-stranded *β*-barrel in the OM of *E. coli* (Ferguson et al., 1998b) (Fig. 1). For a more detailed analysis, we also examined the impact of the N-terminal cork domain on the thermodynamic stability in both environments. A mutant in which the N-terminal cork domain was deleted, FhuAΔ5-160 (Braun, Killmann & Braun, 1999) was isolated after expression into the OM to compare its stabilities in detergent micelles and lipid bilayers with those of wild-type (wt)-FhuA. The thermodynamic stabilities of wt-FhuA and FhuAΔ5-160 were investigated by heat denaturation and by urea-induced unfolding. The effects of pH and of negatively charged phosphatidylglycerol in lipid bilayers were also examined.

## Materials and Methods

### Isolation of FhuA and FhuAΔ5-160

His_6_-tagged wt-FhuA, with the His_6_-tag inserted between residues 405 and 406 of FhuA, was purified from *E. coli* strain AW740 harboring the plasmid pHX405 (Moeck et al., 1996) as described previously. In cells, amino acid 405 of FhuA was found to be surface exposed by flow cytometry and it was, therefore, reasoned that splicing of an His_6_-tag into this position would generate a recombinant FhuA amenable to affinity purification using metal-chelate chromatography (Ferguson et al., 1998a). Similarly, His_6_-tagged FhuAΔ5-160 was isolated from *E. coli* strain BL21 (DE3) carrying plasmid pBk7H (Ferguson et al., 1998b), but with the following modifications: FhuAΔ5-160 solubilized in LDAO-detergent micelles was dialyzed at 6.5 °C against 2 L of ammonium acetate buffer (50 mM, pH 8.0), containing 250 mM NaCl, 5 mM imidazole, and 0.1% LDAO, which is above the CMC of 0.021% (He et al., 2012). FhuA was loaded onto a Ni^2+^-NTA agarose (QIAGEN, Ontario, Canada) column coupled to an automated FPLC (Pharmacia LKB, Uppsala, Sweden). Each variant of FhuA (either wt-FhuA or FhuAΔ5-160) bound selectively to Ni^2+^-NTA agarose and a single symmetrical peak containing purified FhuA eluted at around 200 mM imidazole in a linear imidazole gradient from 5 to 500 mM. The fractions containing purified FhuA were pooled and concentrated by ultrafiltration. Imidazole was removed by dialysis against 2 L of sodium borate buffer (10 mM, pH 9.0) containing 2 mM EDTA and 0.1% LDAO detergent and at 6.5 °C. SDS-PAGE analyses of purified FhuA variants showed single bands at 78 kDa (wt-FhuA) and at 62 kDa (FhuAΔ5-160).

### Reconstitution of wt-FhuA and FhuAΔ5-160 into Phospholipid Bilayers

Phospholipids (Avanti Polar Lipids, Alabaster, AL) were dissolved in chloroform and mixed at the desired ratios. Dry lipid films were prepared under a stream of nitrogen, followed by desiccation for at least 3 h. Lipid films were dispersed in HEPES buffer (10 mM, pH 7.0, containing 2 mM EDTA) followed by addition of FhuA solubilized in LDAO, diluting LDAO below its CMC. Sodium cholate was added to a final concentration of 0.2%. The samples were incubated for 3 h at room temperature. Cholate and LDAO were subsequently removed by 9 h of dialysis against 500 mL of HEPES buffer at 6.5 °C. The dialysis buffer was exchanged every 3 h. Finally, the molar lipid/FhuA ratio was determined as described (Lowry et al., 1951; Rouser et al., 1970) and whenever necessary adjusted by lipid addition. The final concentration of FhuA was 0.64 μM, the lipid concentration was 64 μM. At different pH, HEPES was replaced by citric acid (pH 3), by sodium citrate (pH 4–6), by Tris (pH 8.5), or by borax/sodium hydroxide (pH 10).

### Equilibrium Unfolding

A range of samples of wt-FhuA or FhuAΔ5-160 (1.9 μM), either solubilized in LDAO-detergent micelles at an 800-fold molar excess of LDAO or reconstituted into lipid bilayers, was mixed with buffer containing 10 M urea to obtain various final concentrations of urea ranging from 0 to 8 M in a total volume of 1 mL. Urea is a strong denaturant often used in studies on folding and stability of proteins, including *β*-barrel membrane proteins. To ensure that unfolding of FhuA has reached equilibrium, the time dependence of unfolding was first determined by monitoring the fluorescence signal. All samples were incubated at the selected temperature for at least 24 h prior to recording either fluorescence spectra or circular dichroism spectra. This incubation time was found sufficient for all our experiments, similar as reported for unfolding of FepA from Triton-X-100 detergent (Klug & Feix, 1998; Klug et al., 1995). All spectra were corrected by background subtraction using fluorescence spectra of samples without FhuA, but of otherwise identical composition.

To investigate heat-induced unfolding of wt-FhuA or FhuAΔ5-160, samples were prepared at LDAO/FhuA or phospholipid/FhuA ratios of 800 mol/mol and at an FhuA concentration of 1.9 μM in a total volume of 1 mL buffer. The samples were pre-equilibrated for 24 h at 25 °C. To examine heat-induced unfolding, the temperature was adjusted to 35 °C, followed by an additional equilibration for 3 h before the first fluorescence spectrum was recorded. Subsequently, the temperature was increased by 2 °C followed by an incubation of 20 min, before a new spectrum was recorded. These three steps were repeated until a temperature of 85 °C was reached.

### Fluorescence Spectroscopy

Fluorescence spectra were recorded on a SPEX-Fluorolog-322 spectrometer with double monochromators in the excitation and emission pathways. Tryptophan fluorescence was excited at a wavelength of 290 nm. The excitation and emission bandwidths of the monochromators were 2 nm and 3.7 nm, respectively. Spectra were scanned from 300 to 420 nm; the integration time was 0.05 s; and the increment was 0.5 nm.

### Unfolding Monitored by CD Spectroscopy

Far-UV CD measurements were performed at 30˚C on a Jasco 720 CD spectrometer using a thermostated cuvette (0.1 mm light path). Five scans were accumulated for each spectrum. The scan range was 190 to 250 nm, the response time was 8 s, the bandwidth was 1 nm, and the scan speed was 5 nm/min. The concentration of FhuA was 40 μM. The ratios of LDAO/FhuA or phospholipid/FhuA were 800 mol/mol. Background spectra of samples without FhuA but of otherwise identical composition were subtracted.

### Determination of the Free Energy of Unfolding

Proteins may unfold in one or more steps, which may depend on the number and the stabilities of their domains. Unfolding steps may be treated individually for each domain when they are well resolved and distinguishable from another. For proteins or domains that unfold in one step, a two-state equilibrium may be described. Folded (F) and unfolded (U) states of each domain may coexist at equilibrium in transition regions. The change in free energy per mol protein that unfolds, $\Delta G{^\text{o}_{\rm u}}$, is then obtained by

1 $$\Delta G{^\text{o}_{\rm u}} = - RT\ln \left( K \right) = - RT\ln \left( {\frac{{1 - X_{{\text{F}}} }}{{X_{{\text{F}}} }}} \right)$$

*X*_F_ and *X*_U_ are the mole fractions of the folded and unfolded forms, respectively, *T* is the temperature in Kelvin, *R* the universal gas constant, and *K* the equilibrium constant. Unfolding of membrane proteins like FhuA may be monitored by systematic alteration of experimental parameters leading to unfolding, e.g., as a function of the concentration of a chemical denaturant in solution or as a function of the temperature. For unfolding monitored at increasing concentrations of a chemical denaturant like urea, the effective free energy of unfolding per mol of protein depends linearly on the concentration of the urea in the transition region of unfolding. The free energy in the absence of urea can, therefore, be estimated by extrapolation to 0 M urea, see e.g., refs. (Greene & Pace, 1974; Hong & Tamm, 2004; Klug et al., 1995; Pocanschi et al., 2013; Santoro & Bolen, 1988; Yao & Bolen, 1995):

2 $$\Delta G_{\text{u}}^{\text{o}} (c_{{{\text{urea}}}} ) = \Delta G_{\text{u}}^\text{o} - m \cdot c_{{{\text{urea}}}}$$

$\Delta G{^\text{o}_{\rm u}}$ (*c*_urea_), and $\Delta G{^\text{o}_{\rm u}}$ are the free energies of unfolding in kJ/mol (or kcal/mol) at the concentration *c*_urea_ and extrapolated to *c*_urea_ = 0 M, respectively, and at the other selected experimental standard conditions, e.g., *T*, pH, concentration of buffer, selected environment (detergent micelles, lipid bilayers), and *m* is a measure of cooperativity of unfolding. To obtain *X*_F_ at different concentrations of urea, Eqs. 1 and 2 may be combined to

3 $$X_{{\text{F}}} = \{ exp\left( - {\frac{{\, \Delta G_{\text{u}}^{\text{o}} - m \cdot c_{{{\text{urea}}}} }}{RT}} \right) + 1\}^{ - 1}$$

Concentrations or mole fractions of folded and unfolded forms can be determined by spectroscopy when the spectroscopic signal is a linear combination of the signals of folded and unfolded forms that are proportional to their concentrations. For fluorescence emission spectra of a protein with *f*_*λ*_ as the fluorescence intensity at wavelength *λ*, the intensity-weighted average fluorescence emission maximum $\left\langle \lambda \right\rangle$, given by

4 $$\left\langle \lambda \right\rangle = \frac{{\sum {f_{\lambda } \cdot \lambda } }}{{\sum {f_{\lambda } } }}$$

can be used to relate the fluorescence of a mixture of folded and unfolded protein to the equilibrium constant *K* (Hong & Tamm, 2004; Pocanschi et al., 2006, 2013; Roumestand et al., 2001; Royer et al., 1993). Calculating the sum of fluorescence intensities to obtain $\left\langle \lambda \right\rangle$ has the advantage that the noise typically observed for instrumentally recorded intensities at a selected wavelength is averaged over the entire wavelength range. $\left\langle \lambda \right\rangle$ is, therefore, of higher accuracy than a single-recorded intensity at a selected wavelength. For coexisting folded and unfolded forms, the resulting fluorescence spectrum has an intensity-weighted average fluorescence emission maximum $\left\langle {\lambda_{{\text{M}}} } \right\rangle$ of

5 $$\left\langle {\lambda_{{\text{M}}} } \right\rangle = \frac{{\sum\limits_{\lambda } {\lambda \cdot F_{\lambda } \left( {{\text{F}} + {\text{U}}} \right)} }}{{\sum\limits_{\lambda } {F_{\lambda } \left( {{\text{F}} + {\text{U}}} \right)} }} = \frac{{X_{{\text{F}}} \cdot \sum\limits_{\lambda } {\lambda \cdot f_{\lambda } \left( {\text{F}} \right)} + \left( {1 - X_{{\text{F}}} } \right) \cdot \sum\limits_{\lambda } {\lambda \cdot f_{\lambda } \left( {\text{U}} \right)} }}{{X_{{\text{F}}} \cdot \sum\limits_{\lambda } {f_{\lambda } \left( {\text{F}} \right)} + \left( {1 - X_{{\text{F}}} } \right) \cdot \sum\limits_{\lambda } {f_{\lambda } \left( {\text{U}} \right)} }}$$

*F*_*λ*_(F + U) at wavelength *λ* is a linear combination of the fluorescence signals *f*_*λ*_(F) and *f*_*λ*_(U) of the folded and unfolded forms, respectively, at wavelength *λ* present in the sample at molar fractions *X*_F_ and *X*_U_, respectively. Defining $\left\langle {\lambda_{{\text{F}}} } \right\rangle$and $\left\langle {\lambda_{{\text{U}}} } \right\rangle$ as the $\left\langle \lambda \right\rangle$ of the fluorescence spectra of the folded and unfolded forms of the protein, respectively, Eq. 5 can be rewritten to obtain the mole fraction of the folded protein, *X*_F_:

6 $$X_{{\text{F}}} = \left( {\frac{{\left\langle {\lambda_{{\text{M}}} } \right\rangle - \left\langle {\lambda_{{\text{F}}} } \right\rangle }}{{\left\langle {\lambda_{{\text{U}}} } \right\rangle - \left\langle {\lambda_{{\text{M}}} } \right\rangle }} \cdot \frac{{\sum\limits_{\lambda } {f_{\lambda } \left( {\text{F}} \right)} }}{{\sum\limits_{\lambda } {f_{\lambda } \left( {\text{U}} \right)} }} + 1} \right)^{ - 1}$$

From *X*_F_, the equilibrium constant of unfolding *K* = (1–*X*_F_) / *X*_F_ is obtained:

7 $$K = \frac{{\left\langle {\lambda_{{\text{F}}} } \right\rangle - \left\langle {\lambda_{{\text{M}}} } \right\rangle }}{{\left\langle {\lambda_{{\text{M}}} } \right\rangle - \left\langle {\lambda_{{\text{U}}} } \right\rangle }} \cdot \frac{{\sum\limits_{\lambda } {f_{\lambda } \left( {\text{F}} \right)} }}{{\sum\limits_{\lambda } {f_{\lambda } \left( {\text{U}} \right)} }} = \frac{{\left\langle {\lambda_{{\text{F}}} } \right\rangle - \left\langle {\lambda_{{\text{M}}} } \right\rangle }}{{\left\langle {\lambda_{{\text{M}}} } \right\rangle - \left\langle {\lambda_{{\text{U}}} } \right\rangle }} \cdot \phi_{{\text{R}}}$$

with

$$\phi_{{\text{R}}} = \frac{{\sum\limits_{\lambda } {f_{\lambda } \left( {\text{F}} \right)} }}{{\sum\limits_{\lambda } {f_{\lambda } \left( {\text{U}} \right)} }}$$

$\left\langle {\lambda_{{\text{M}}} } \right\rangle$ is then obtained from Eqs. (6) and (7) to

8 $$\left\langle {\lambda_{\text{M}} } \right\rangle = \frac{{\left\langle {\lambda_{\text{F}} } \right\rangle + \frac{1}{{\phi_{\text{R}} }}K\left\langle {\lambda_{\text{U}} } \right\rangle }}{{\left( {1 + \frac{1}{{\phi_{\text{R}} }}K} \right)}}$$

Intensity-weighted average emission maxima of fluorophores $\left\langle {\lambda } \right\rangle$ depend on the solvent. Experimentally, linear dependencies of $\left\langle {\lambda_{\text{F}} } \right\rangle$ and $\left\langle {\lambda_{{\text{U}}} } \right\rangle$ on the concentration of the denaturant urea are observed (Santoro & Bolen, 1988), with $\left\langle {\lambda_{{\text{U}}} } \right\rangle = \left\langle {\lambda_{{\text{U}}} } \right\rangle_{0} + m_{\text{F}} \cdot c_{{{\text{urea}}}}$ and $\left\langle {\lambda_{{\text{F}}} } \right\rangle = \left\langle {\lambda_{{\text{F}}} } \right\rangle_{0} + m_{\text{F}} \cdot c_{{{\text{urea}}}}$, where the index 0 indicates the absence of urea, whereas *m*_F_ and *m*_U_ are the slopes of the linear dependencies of $\left\langle {\lambda } \right\rangle$ on the concentration of urea *c*_urea_. Equations (1), (2), and (8) can be combined to obtain the standard free energy of unfolding (Santoro & Bolen, 1988):

9 $$\langle \lambda_{{\text{M}}} \rangle = \frac{{\left( {\langle \lambda_{{\text{F}}} \rangle_{0} + m_{{\text{F}}} \cdot c_{{{\text{urea}}}} } \right) + \left( {\langle \lambda_{{\text{U}}} \rangle_{0} + m_{{\text{U}}} \cdot c_{{{\text{urea}}}} } \right) \cdot \frac{1}{{\phi_{{\text{R}}} }} \cdot exp\left( { - \frac{{\Delta G_{{\text{u}}}^{\text{o} } - m \cdot c_{{{\text{urea}}}} }}{RT}} \right)}}{{1 + \frac{1}{{\phi_{{\text{R}}} }} \cdot exp\left( { - \frac{{\Delta G_{{\text{u}}}^{\text{o} } - m \cdot c_{{{\text{urea}}}} }}{RT}} \right)}}$$

Equation 9 was fitted to the $\left\langle {\lambda_{{\text{M}}} } \right\rangle$ obtained from the fluorescence spectra at the various concentrations of urea used to examine unfolding of FhuA using IGOR Pro (Wavemetrics, Oregon). The extrapolated free energy of unfolding in the absence of the denaturant, $\Delta G{^\text{o}_{\rm u}}$,﻿ and the slope *m* that describes the change in the free energy as a function of the denaturant concentration (*c*_urea_), were free fit parameters. The ratio $\phi_\text{R}$﻿ of the total fluorescence intensities of the folded and unfolded forms of FhuA was determined from the respective fluorescence spectra and used as a fixed fit parameter.

### Temperature Dependence of the Free Energy of Unfolding and Enthalpy of Unfolding

The temperature dependence of $\Delta G{^\text{o}_{\rm u}}$ is described by a modified form of the Gibbs–Helmholtz equation Δ*G*°(*T*) = Δ*H*° – *T*Δ*S*° and can be obtained from the unfolding transition at the protein melting temperature *T*_M_, *i.e.,* at the midpoint of the thermal unfolding transition, *cf.* (Becktel & Schellman, 1987; Elwell & Schellman, 1977):

10 $$\Delta G^{\text{o} } \left( T \right) = \Delta H_{{\text{M}}}^{\text{o} } \cdot \left( {1 - \frac{T}{{T_{{\text{M}}} }}} \right) + \Delta c_{{\text{P}}} \left[ {T - T_{{\text{M}}} - T \cdot \ln \left( {\frac{T}{{T_{{\text{M}}} }}} \right)} \right]$$

In Eq. (10), Δ*G°*(*T*) is $\Delta G{^\text{o}_{\rm u}}$ at a temperature *T* and Δ*c*_p_ is the change in heat capacity of the protein upon unfolding. *T*_M_ is the unfolding temperature and $\Delta H{_{\rm M}^\text{o}}$ the change of the enthalpy at the unfolding temperature.

## Results

### Fluorescence Spectroscopy Indicates Urea-Induced Unfolding of Detergent Solubilized FhuA

**Fig. 2:**
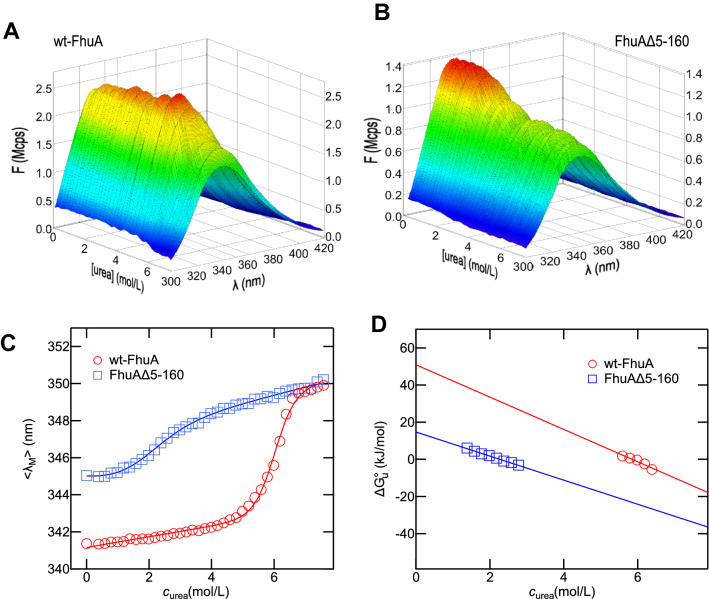
Fluorescence data indicated that removal of the N-terminal cork domain destabilizes FhuA solubilized in LDAO-detergent micelles. Fluorescence spectra of (**A**) wt-FhuA and (**B**) FhuAΔ5-160 recorded in the wavelength range from 300 to 420 nm at 30 °C. Samples contained 0.64 μΜ FhuA and 4.4 mM LDAO in 10 mM HEPES buffer (pH 7) and urea at the concentration indicated. (**C**) The intensity-weighted average fluorescence emission maxima $\left\langle {\lambda_{{\text{M}}} } \right\rangle$ of wt-FhuA (red circle) and FhuAΔ5-160 (blue square) were calculated from the spectra and plotted as a function of the urea concentration. $\left\langle {\lambda_{{\text{M}}} } \right\rangle$ was shifted toward longer wavelengths when the concentration of urea in the sample exceeded ~ 5 M for wt-FhuA and about ~ 1 M urea for FhuAΔ5-160. To estimate the effective free energies of unfolding of FhuA (Δ*G*°), Eq. 9 was fitted to the unfolding data (solid lines). (**D**) Δ*G*° was calculated at the different urea concentrations in the transition regions of unfolding of wt-FhuA (red circles) or FhuAΔ5-160 (blue squares), respectively and depended linearly on the urea concentration

To investigate and compare the thermodynamic stabilities of wt-FhuA (Fig. 1A-C) and FhuAΔ5-160 (Fig. 1D) in lipid bilayers and in detergent micelles, both were extracted in native form from the OM of *E. coli* into micelles of the detergent LDAO in aqueous buffer. We first examined unfolding of wt-FhuA and FhuAΔ5-160 by Trp fluorescence spectroscopy, which is a sensitive tool to monitor conformational changes of proteins, as the fluorescence intensity and the wavelength of the maximum of the spectrum depend directly on the molecular environment of the fluorescent tryptophan side chains, which is different for folded and unfolded forms. Unfolding of FhuA was induced at increased urea concentrations. The spectra recorded for wt-FhuA (Fig. 2A) and for FhuAΔ5-160 (Fig. 2B) were used to calculate $\left\langle {\lambda_{{\text{M}}} } \right\rangle$. A graph of $\left\langle {\lambda_{{\text{M}}} } \right\rangle$ as a function of *c*_urea_ indicated that from LDAO micelles, FhuA unfolded in a single transition (Fig. 2C). This was observed for both wt-FhuA and FhuAΔ5-160 and indicated that unfolding of the cork and of the *β*-barrel domain were coupled when wt-FhuA was solubilized in LDAO-detergent micelles. The transitions of wt-FhuA and FhuAΔ5-160 were both characterized by a decrease of the fluorescence intensity relative to the intensity observed in the absence of urea and by a shift of $\left\langle {\lambda_{{\text{M}}} } \right\rangle$ toward longer wavelength (Fig. 2C). For wt-FhuA, this shift was $\Delta\left\langle{\lambda_{{\text{M}}} } \right\rangle$ ~ 9 nm, from Δ$\left\langle {\lambda_{{\text{M}}} } \right\rangle$ ~ 341 nm in the absence of urea to Δ$\left\langle {\lambda_{{\text{M}}} } \right\rangle$ ~ 350 nm in the presence of 8 M urea. For FhuAΔ5-160, the shift was Δ$\left\langle {\lambda_{{\text{M}}} } \right\rangle$ ~  + 5 nm from $\left\langle {\lambda_{{\text{M}}} } \right\rangle$ ~ 345 nm in the absence to Δ$\left\langle {\lambda_{{\text{M}}} } \right\rangle$ ~ 350 nm in the presence of 8 M urea. Differences of the Δ$\left\langle {\lambda_{{\text{M}}} } \right\rangle$ of wt-FhuA and FhuAΔ5-160 in LDAO at 0 M urea were likely caused by tryptophan 21, present in wt-FhuA, but not in FhuAΔ5-160.

The single transitions from the folded to the unfolded forms were observed between 1 and 3 M urea for FhuAΔ5-160 and between 5 and 7 M urea for wt-FhuA, indicating that removal of the cork domain (residues 1–160) strongly decreases the stability of FhuA. The midpoints of unfolding were determined to 6 M urea for wt-FhuA (○) and to 2.2 M urea for FhuAΔ5-160 (□). To analyze unfolding of FhuA from LDAO micelles in buffer, the intensity-weighted average fluorescence emission maxima $\left\langle {\lambda_{{\text{M}}} } \right\rangle$ were calculated (Eq. 4) and plotted as a function of the concentration of urea (Fig. 2C). For a wide range of soluble proteins (Pace & Shaw, 2000) and also other *β*-barrel membrane proteins like FepA (Klug et al., 1995) and OmpA (Hong & Tamm, 2004; Pocanschi et al., 2006, 2013), it was shown that the molar-free energy of unfolding depends linearly on the urea concentration in the transition region, where folded and unfolded forms of a protein coexist.

It should be noted that for membrane proteins, a standard state is not readily defined as there is a great variation among the membrane environments and the molar-free energy of folding or unfolding depends on many parameters, like lipid-protein interactions, overall membrane properties, pH, etc. For simplicity, the symbol $\Delta G{^\text{o}_{\rm u}}$ is used here to describe the effective change in free energy upon unfolding of 1 mol protein under the selected conditions of the experiment to distinguish it from the system-extensive Δ*G*, which is zero at equilibrium. The linear dependence of $\Delta G{^\text{o}_{\rm u}}$ on the concentration of urea was also confirmed for wt-FhuA and FhuAΔ5-160 when the effective free energies in the transition regions of FhuA unfolding were calculated from the corresponding spectra using Eqs. 1 and 6 and plotted vs. *c*_urea_ (Fig. 2D). Here, the free energies of unfolding extrapolated to *c*_urea_ = 0 M were calculated by fitting Eq. 9 to the entire data from 0 to 8 M urea (Fig. 2C, solid lines), leading to $\Delta G{^\text{o}_{\rm u}}$ ~ 46 kJ/mol for wt-FhuA and to $\Delta G{^\text{o}_{\rm u}}$ ~ 6 kJ/mol for FhuAΔ5-160.

### CD Spectroscopy Confirms a Two-State Unfolding of Detergent-Micelle-Solubilized FhuA

**Fig. 3:**
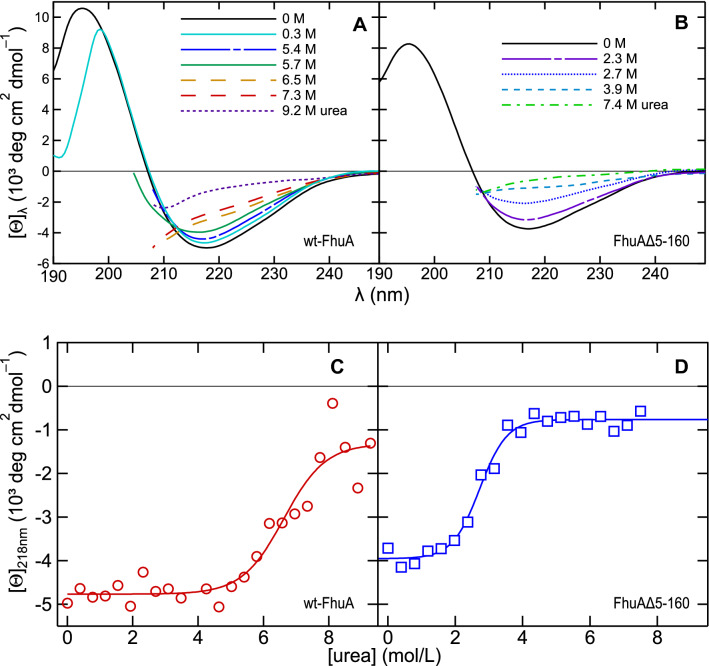
Circular dichroism spectroscopy indicated the *β*-sheet secondary structure of the *β*-barrel domain is less stable upon removal of the cork domain. Circular dichroism spectra of (**A**) wt-FhuA and of (**B**) FhuAΔ5-160 were recorded at various concentrations of urea. Urea strongly absorbed UV light and led to very high noise levels in the CD signal at wavelengths of 208 nm or shorter. Therefore, at concentrations greater than ~ 0.5 M urea, ellipticities could reliably be recorded only at λ >  ~ 208 nm. (**C**) and (**D**) Unfolding of the *β*-sheet secondary structure of solubilized wt-FhuA (C) and FhuAΔ5-160 (D) as determined from ellipticities recorded at 218 nm and at 30 °C as a function of the concentration of urea. In all samples, the concentration of FhuA was 40 μM at a molar LDAO/FhuA ratio of 800. FhuA was solubilized in LDAO in 10 mM HEPES buffer, and 2 mM EDTA at pH 7

To confirm the loss of the *β*-barrel structure of LDAO-solubilized FhuA by urea-induced unfolding, we recorded circular dichroism (CD) spectra (Fig. 3, A and B). The CD spectra of wt-FhuA (Fig. 3A) and FhuAΔ5-160 (Fig. 3B) in the absence or at low concentrations of urea showed a single minimum between 215 and 218 nm with spectral amplitudes of ~ –5000 and ~ –4000 deg cm^2^ dmol^–1^, respectively, and a single maximum at ~ 195 nm. The line shapes were characteristic for folded *β*-barrel membrane proteins. At higher concentrations, urea strongly absorbs UV radiation below 208 nm, and therefore, CD spectra could only be recorded above ~ 205 nm. The reduction of the absolute intensities of the CD spectra and the disappearance of the relative minima between 208 and 250 nm at the higher concentrations of urea were characteristic for a loss of *β*-sheet secondary structure. This loss of secondary structure was observed for both FhuA variants at high urea concentrations. The dependence of the spectral line shape and amplitude at 218 nm on the concentration of denaturant showed that wt-FhuA loses its *β*-sheet structure at much higher concentration of urea than FhuAΔ5-160. The change of the CD signal at 218 nm plotted as a function of the urea concentration for both wt-FhuA (Fig. 3C) and FhuAΔ5-160 (Fig. 3D) indicated a two-state unfolding of FhuA from LDAO micelles. The midpoints were at 6.2 M urea for wt-FhuA and at 2.7 M urea for FhuAΔ5-160. The urea concentrations, at which FhuA unfolded were very similar to those observed by fluorescence spectroscopy (Fig. 2C) and confirmed the two-state unfolding process with either all or no *β*-sheet structure present in FhuA. The results also indicated that the cork domain, emphasized in black in the crystal structure of wt-FhuA (Fig. 1), does not unfold independently from the *β*-barrel domain of wt-FhuA when solubilized in LDAO micelles, likely because it is connected to the *β*-barrel domain by extensive hydrogen bonding (Ferguson et al., 1998b; Locher et al., 1998).

### The Stability of FhuA Is Strongly Temperature Dependent

**Fig. 4:**
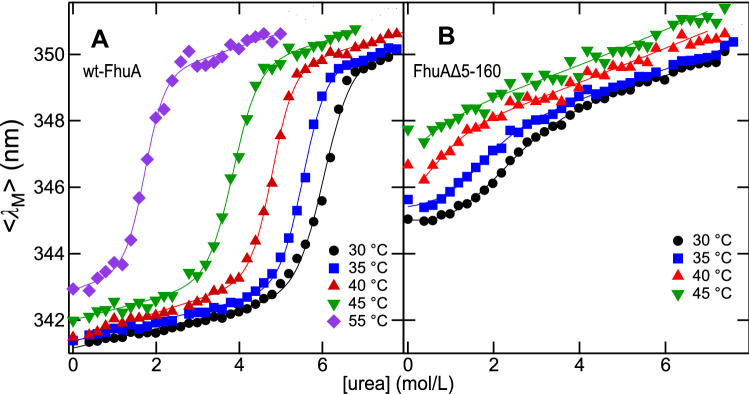
Urea-induced unfolding of FhuA is strongly temperature dependent. (**A**) wt-FhuA and (**B**) FhuAΔ5-160 were incubated at various concentrations of urea at 30 °C (●), 35 °C (blue square), 40 °C (Red triangle), 45 °C (green down pointing triangle), and 55 °C (violet diamond) to monitor their unfolding from LDAO micelles by fluorescence spectroscopy as described in the legend to Fig. 2 for 30 °C. The intensity-weighted average fluorescence emission maxima $\left\langle {\lambda_{{\text{M}}} } \right\rangle$ were calculated from the spectra and plotted as a function of the urea concentration, ranging from 0 to 8 M. Equation 9 was fitted to these data at each temperature (solid lines). FhuA (0.64 μM) was present in 4.4 mM LDAO in 10 mM HEPES Buffer at pH 7 at the urea concentration indicated

The stability of FhuA against urea-induced unfolding was further examined at 35, 40, 45, and 55 °C. Unfolding was monitored by fluorescence spectroscopy and the dependence of $\left\langle {\lambda_{{\text{M}}} } \right\rangle$ on the concentration of urea at the selected temperatures for samples containing either wt-FhuA (Fig. 4A) or FhuAΔ5-160 (Fig. 4B) solubilized in LDAO micelles was plotted. Unfolding of wt-FhuA was characterized by midpoints of the unfolding curves at 6 M (30 °C), 5.6 M (35 °C), 4.8 M (40 °C), 3.8 M (45 °C) and 1.7 M (55 °C) urea, indicating an increased destabilization of wt-FhuA with temperature. FhuAΔ5-160 was again much less stable than wt-FhuA, with midpoints of the unfolding curves at 2.2 M (30 °C) and 1.5 M (35 °C) urea. To determine the effective free energies of unfolding, we fitted Eq. 9 to the experimental data. The free energies of unfolding and the corresponding *m*-values are listed in Table 1. wt-FhuA lost more than half of its stability when the temperature was raised from 30 °C to 55 °C. At any selected temperature, FhuAΔ5-160 was far less stable than wt-FhuA, confirming the stabilization of wt-FhuA by the cork domain when solubilized in LDAO micelles.

**Table 1:** $\Delta G{^\text{o}_{\rm u}}$ and *m*-values estimated for unfolding of LDAO-solubilized FhuA

	Temperature (°C)	$\Delta G{^\text{o}_{\rm u}} \text{(kJ/mol)}$	*m*-value (kJ/mol)
**A** wt-FhuA in LDAO micelles			
	30	46 ± 2	7.1 ± 0.6
	35	45 ± 2	8.6 ± 0.5
	40	45 ± 2	9.5 ± 0.5
	45	35 ± 1	8.8 ± 0.7
	55	19 ± 1	10.6 ± 1.7

	Temperature (°C)	$\Delta G{^\text{o}_{\rm u}} \text{(kJ/mol)}$	*m*-value (kJ/mol)
**B** FhuAΔ5-160 in LDAO micelles			
	30	6 ± 2	3.5 ± 0.4
	35	5 ± 3	4.4 ± 0.8

$\Delta G{^\text{o}_{\rm u}}$ and *m*-values of unfolding of wt-FhuA (A) and FhuAΔ5-160 (B), solubilized by LDAO (at an 800-fold molar excess) in 10 mM HEPES Buffer (pH 7) were obtained from non-linear least-square fits of Eq. 9 to the unfolding experiments shown in Fig. 4

### Heat-Induced Unfolding of FhuA Confirms Stabilization of FhuA by Its Cork Domain

**Fig. 5:**
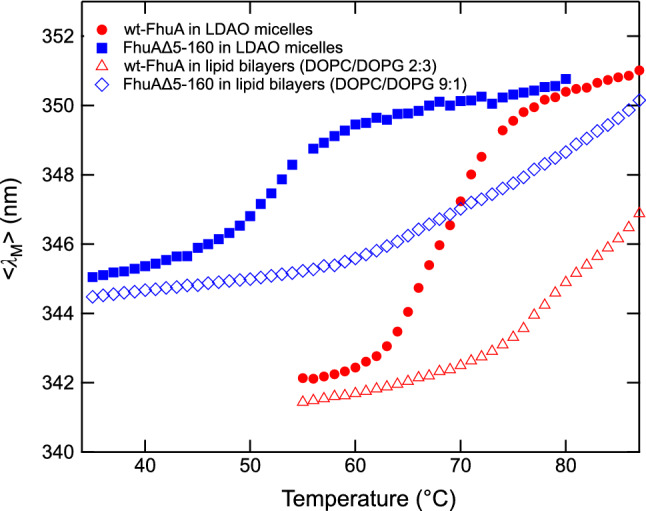
In comparison to an LDAO-detergent-micelle environment, reconstitution into lipid bilayers strongly stabilizes FhuA. The cork domain stabilizes wt-FhuA against heat-induced unfolding from LDAO micelles. Heat-induced unfolding of FhuA was monitored by recording fluorescence spectra of wt-FhuA and FhuAΔ5-160 as a function of temperature. Spectra were recorded starting at 35 °C. The temperature was then raised in steps of 1 °C, and after an incubation time of 20 min at each temperature, additional spectra were recorded until a temperature of 87 °C was reached. The intensity-weighted average emission maxima $\left\langle {\lambda_{{\text{M}}} } \right\rangle$ of the recorded spectra are shown as a function of temperature for wt-FhuA (red circle) and FhuAΔ5-160 (blue square) in LDAO micelles, of wt-FhuA in lipid bilayers of DOPC/DOPG 2:3 (red triangle), and of FhuAΔ5-160 in bilayers of DOPC/DOPG 9:1 (blue diamond). The FhuA concentration was 1.9 μM at a molar lipid/FhuA ratio of 800 in all samples

The data shown in Fig. 4 indicated that the stabilities of both forms of FhuA are strongly temperature dependent. We next examined the heat-induced unfolding of the two forms solubilized in LDAO micelles directly by recording fluorescence spectra of FhuA in the absence of urea at various temperatures from 35 °C to ~ 90 °C. Figure 5 shows the dependence of $\left\langle {\lambda_{{\text{M}}} } \right\rangle$ of the fluorescence spectra on temperature for FhuAΔ5-160 (blue square) and for wt-FhuA (red circle). Unfolding of wt-FhuA from LDAO micelles was characterized by a temperature of the midpoint of unfolding (*T*_M,_) at *T*_M_ ~ 69 °C that is ~ 17 °C above the *T*_M_ ~ 52 °C of FhuAΔ5-160. This confirmed the strong stabilization of FhuA by its cork domain, previously observed for the urea-induced unfolding processes of the two forms.

### Temperature Dependence of the Energy of Unfolding of wt-FhuA and FhuAΔ5-160

**Fig. 6:**
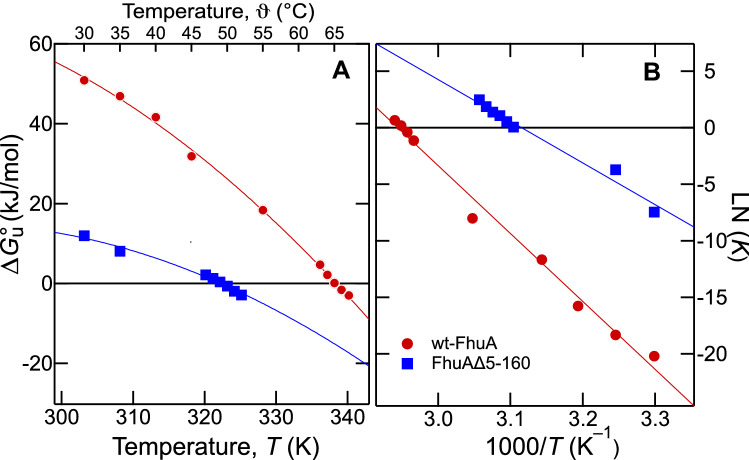
**A** The effective free energies $\Delta G{^\text{o}_{\rm u}}$ of unfolding of wt-FhuA (red circle) and FhuAΔ5–160 (blue square) solubilized in LDAO decrease with temperature. Values of $\Delta G{^\text{o}_{\rm u}}$ at different temperatures were calculated by fitting Eq. 9 to the urea unfolding experiments shown in Fig. 4 and from the heat denaturation experiments shown in Fig. 5. Equation (10) (solid lines) was then fitted to the $\Delta G{^\text{o}_{\rm u}}$ ﻿obtained at the different temperatures. **B** The van’t Hoff plot of the data from panel A shows a linear dependence of LN(K) on the reciprocal temperature

The temperature dependence of the free energy of unfolding can be used to calculate enthalpy and entropy of unfolding (Becktel & Schellman, 1987; Pace et al., 1999). Analysis of the temperature dependence of the effective free energies of unfolding of wt-FhuA (red circle) and FhuAΔ5-160 (dark blue square), each solubilized in LDAO micelles (Fig. 6A) was performed for both, urea-induced unfolding of FhuA (Fig. 4), using Eq. 9, and heat-induced unfolding of FhuA (Fig. 5) using Eqs. 1 and 6. The van’t Hoff plots of ln(*K*) vs. 1/*T* were linear (Fig. 6B) for wt-FhuA (red circle) and for FhuAΔ5-160 (dark blue square). From fits of Eq. 10 (solid lines) to the effective free energies as a function of temperature (Fig. 6A), enthalpies were estimated to $\Delta H{_{\rm M}^\text{o}}$﻿ ~ 250 kJ/mol for FhuAΔ5-160 and to $\Delta H{_{\rm M}^\text{o}}$﻿ ~ 640 kJ/mol for wt-FhuA. The corresponding melting temperatures *T*_M_, entropies $\Delta S{_{\rm M}^\text{o}}$, and changes in heat capacities Δ*c*_P_ of wt-FhuA and FhuAΔ5-160 are listed in Table 2.

**Table 2:** Enthalpies, Entropies, and temperatures obtained from unfolding experiments of FhuA, FhuAΔ5-160, and OmpA after solubilization in LDAO micelles

	*T*_M_(°C)	$\Delta H{_{\rm M}^\text{o}} (\text{kJ/mol})$	$\Delta S{_{\rm M}^\text{o}} (\text{kJ/mol})$	$\Delta c_{\text{p}}$ (kJ mol^−1^ K^−1^)
wt-FhuA	65.3 ± 0.2	643 ± 28	1.9	8.2 ± 2.8
FhuAΔ5-160	49.1 ± 0.4	255 ± 40	0.8	6.5 ± 4.7

Estimates are based on non-linear least-square fits of Eq. 10 to the data shown in Fig. 6a. At *T*_M_, $\Delta G{^\text{o}_{\rm u}}$ = 0 (Fig. 6) and $\Delta S{_{\rm M}^\text{o}}$ can be obtained from $\Delta G{^\text{o}_{\rm u}}$ = $\Delta G{^\text{o}_{\rm u}}=\Delta H{_{\rm M}^\text{o}}-T \cdot \Delta S{_{\rm M}^\text{o}}$

### The Lipid Bilayer Strongly Stabilizes FhuA and FhuAΔ5-160 Against Heat-Induced Unfolding

From many studies on the functions of TMPs, it is known that detergent-micelle extraction of membrane proteins often leads to their inactivation (Champeil et al., 2016; Miles et al., 2011; Zhou et al., 2001). To determine differences in the stability of transmembrane proteins like FhuA in LDAO-detergent-micelle vs. phospholipid bilayer environments, we reconstituted LDAO-solubilized FhuA into bilayers containing dioleoyl phosphatidylcholine (DOPC) and dioleoyl phosphatidylglycerol (DOPG). Heat-induced unfolding of both forms was examined by fluorescence spectroscopy, and the $\left\langle {\lambda_{{\text{M}}} } \right\rangle$ of the spectra were again plotted as a function of temperature. Lipid bilayer-inserted wt-FhuA (Fig. 5, red triangle) or FhuAΔ5-160 (Fig. 5, white blue diamond) was heated to temperatures as high as the aqueous buffer allowed (~ 90 °C), but unfolding was still incomplete when approaching the boiling point. Therefore, we were not able to obtain fluorescence spectra of the completely heat-denatured FhuA when reconstituted into lipid bilayers. This was in contrast to LDAO-solubilized wt-FhuA or FhuAΔ5-160 that completely denatured above 60 °C and 75 °C, respectively. Taking $\left\langle {\lambda_{{\text{M}}} } \right\rangle$ values of unfolded wt-FhuA and unfolded FhuAΔ5-160 that were obtained by urea-induced unfolding and fits of Eq. 9 to data shown in Fig. 4, it was still possible to crudely estimate the temperatures at the midpoints of unfolding (*T*_M_) of both variants of FhuA for their inserted and folded forms in lipid bilayers. A *T*_M_ of ~ 70 °C was obtained for FhuAΔ5-160, reconstituted into bilayers of DOPC and DOPG at a molar ratio of 9:1, and a *T*_M_ ~ 85 to 90 °C was estimated for wt-FhuA after reconstitution into bilayers of DOPC and DOPG at a molar ratio of 2:3. The stabilities of wt-FhuA and FhuAΔ5-160 cannot be directly compared as the compositions of the lipid bilayers are different. However, unfolding of either wt-FhuA or FhuAΔ5-150 from LDAO micelles may be used as a common basis to examine stabilization of wt-FhuA and FhuAΔ5-150 by lipid bilayers. The midpoints of unfolding of wt-FhuA and FhuAΔ5-160 in lipid bilayers were both observed at temperatures that were Δ*T*_M_ ~ 18 to 21 °C higher than observed for the corresponding LDAO-micelle-solubilized wt-FhuA and FhuAΔ5-160. The large Δ*T*_M_ indicated the stabilities of both, wt-FhuA and FhuAΔ5-160 were strongly increased regardless of the lipid composition of the bilayer, reflecting strong differences between the physical properties of micelles and bilayers. For direct comparisons of the stabilities of wt-FhuA and FhuAΔ5-160, each reconstituted in bilayers of DOPC/DOPG at a ratio of 2:3 and for comparisons of the stabilities of FhuAΔ5-160 in bilayers composed of DOPC and DOPG either at a ratio of 9:1 or at a ratio of 2:3, see below.

### wt-FhuA Unfolds from Lipid Bilayers in Two Steps

The stability of FhuA in lipid bilayers was more closely examined by monitoring its resistance against unfolding at increased concentrations of urea. Several unfolding experiments were performed at selected temperatures between 35 °C and 55 °C and monitored by fluorescence spectroscopy. In comparison to its LDAO-solubilized form, the midpoints of the unfolding transitions of FhuAΔ5-160 in bilayers of DOPC and DOPG at a molar ratio of 2:3 were shifted toward higher urea concentrations, namely 5.8 M (35 °C), 5.1 M (40 °C), 4.3 M (45 °C), and 1.4 M (55 °C) urea. Comparisons with unfolding experiments of FhuAΔ5-160 in micelles of lyso-lauroylphosphatidylcholine or octadecanoyldimethylammonium-N-oxide confirmed that the lipid bilayer has a stabilizing effect on FhuAΔ5-160 when compared to detergent micelles (data not shown).

**Fig. 7:**
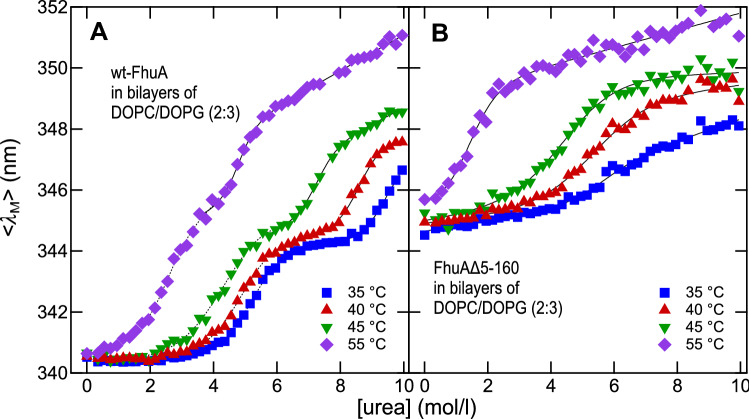
Reconstituted into lipid bilayers, wt-FhuA but not FhuAΔ5-16 displays a three-state unfolding. Unfolding of (**A**) wt-FhuA and (**B**) FhuAΔ5-160 with urea was performed after reconstitution of FhuA into phospholipid bilayers composed of DOPG and DOPC at a molar ratio of 3:2. Fluorescence spectra of FhuA were recorded and the intensity-weighted average emission maxima $\left\langle {\lambda_{{\text{M}}} } \right\rangle$ were plotted as a function of the urea concentration. Unfolding experiments were performed at 35 °C (blue square), 40 °C (red triangle), 45 °C (green down pointing triangle), and at 55 °C (violet diamond). Equation 9 was fitted to each dataset (solid lines). The concentration of FhuA was 0.64 μM, the lipid concentration was 64 μM

In contrast to unfolding of the solubilized forms of FhuA from LDAO micelles and in contrast to unfolding of FhuAΔ5-160 from lipid bilayers, wt-FhuA unfolded in two steps from lipid bilayers (Fig. 7A). In addition to the unfolded and folded states, an intermediate third state of wt-FhuA was observed at all temperatures. At 40 °C, the first unfolding step of wt-FhuA was characterized by an increase of $\left\langle {\lambda_{{\text{M}}} } \right\rangle$ from ~ 340.5 to ~ 344 nm. In a second step, $\left\langle {\lambda_{{\text{M}}} } \right\rangle$ increased from ~ 344 to ~ 347.6 nm. At this temperature, unfolding of FhuAΔ5-160 was characterized by a single transition with a change in $\left\langle {\lambda_{{\text{M}}} } \right\rangle$ from 344 to 349 nm (Fig. 7B), which correlated well with the second stage of wt-FhuA unfolding (Fig. 7A). The single Trp-21 that is present in the N-terminal cork domain has a strongly reduced fluorescence intensity when this domain is unfolded and fluorescence of Trp-21 is then shifted toward longer wavelengths. The contribution of the stronger fluorescent 8 Trps in the folded *β*-barrel domain then dominates overall fluorescence properties, which were similar to those of folded FhuAΔ5-160. In contrast to detergent solubilized wt-FhuA, the lipid membrane stabilized the *β*-barrel domain to an extent that the cork domain can unfold without a concurrent denaturation of the *β*-barrel domain (Fig. 7A). In lipid bilayers, but not in detergent, unfolding of the N-terminal cork domain of wt-FhuA is uncoupled from unfolding of its *β*-barrel domain.

The free energies of unfolding obtained from fits of Eq. 9 to the observed unfolding of FhuA in bilayers are listed in Table 3. In comparison to unfolding from detergent micelles, FhuAΔ5-160 was about three to four times more stable in lipid bilayers ($\Delta G{^\text{o}_{\rm u}}$ ~ 21 ± 6 kJ/mol at 35 °C, pH 7) than in LDAO micelles ($\Delta G{^\text{o}_{\rm u}}$﻿ ~ 5 ± 3 kJ/mol at 35 °C, pH 7). The two steps of unfolding of wt-FhuA from bilayers were characterized by $\Delta G{^\text{o}_{\rm u, 1}}$ ~ 25 ± 2 kJ/mol and $\Delta G{^\text{o}_{\rm u, 2}}$ ~ 57 ± 14 kJ/mol (at 40 °C, pH 7). Summed, $\Delta G{^\text{o}_{\rm u}}$ was ~ 83 ± 16 kJ/mol for the complete unfolding process of wt-FhuA from lipid bilayers containing DOPC and DOPG at a ratio of 2:3. In comparison to the LDAO-solubilized wt-FhuA, the bilayer-integrated wt-FhuA was about twice as stable. While the difference between the stabilities of wt-FhuA and FhuAΔ5-160 in LDAO micelles caused by the cork domain was ~ 40 kJ/mol at 30 or 35 °C (Table 1), a difference of ~ 48 to 60 kJ/mol, depending on temperature, was found when FhuA was present in lipid bilayers and when the $\Delta G{^\text{o}_{\rm u}}$ of the transitions of both domains of wt-FhuA were taken together (Table 3).

**Table 3:** $\Delta G{^\text{o}_{\rm u}}$ and *m*-values estimated for unfolding of FhuA from lipid bilayers

	Temperature (°C)	$\Delta G{^\text{o}_{\rm u}} \text{(kJ/mol)}$	*m*-value (kJ/mol)
**A** wt-FhuA^a^			
(1. transition)	35	25 ± 2	4.8 ± 0.3
	40	20 ± 1	4.3 ± 0.4
	45	13 ± 2	3.2 ± 0.6
	55	14 ± 4	6.0 ± 2
(2. transition)	35	59 ± 12	6.3 ± 1.2
	40	57 ± 14	6.6 ± 1.7
	45	55 ± 13	7.7 ± 1.7
	55	41 ± 10	8.7 ± 1.9

	Temperature (°C)	$\Delta G{^\text{o}_{\rm u}} \text{(kJ/mol)}$	*m*-value (kJ/mol)
**B** FhuAΔ5-160			
	35	21 ± 6	3.9 ± 1.1
	40	16 ± 4	3.0 ± 0.6
	45	12 ± 3	3.8 ± 0.6
	55	7 ± 4	5.6 ± 1.3

^a^$\Delta G{^\text{o}_{\rm u}}$ ﻿and *m*-values of unfolding of wt-FhuA (A) and and FhuAΔ5-160 (B) after reconstitution into lipid bilayers composed of composed DOPC and DOPG at a molar ratio of 2:3 in 10 mM HEPES Buffer (pH 7). Estimates were obtained from non-linear least-square fits of Eq. 9 to the unfolding data shown in Fig. 7

From the temperature dependence of the free energy of unfolding of FhuA, the enthalpies may be roughly estimated to $\Delta H{_{\rm M}^\text{o}}$ ~ 355 kJ/mol for the first step of unfolding and to $\Delta H{_{\rm M}^\text{o}}$ ~ 632 kJ/mol for the second step of unfolding. For FhuAΔ5-160, $\Delta H{_{\rm M}^\text{o}}$﻿ was ~ 342 kJ/mol.

### FhuAΔ5-160 Reconstituted into Lipid Bilayers Is Most Stable at Mildly Acidic pH 4 to pH 6

**Fig. 8:**
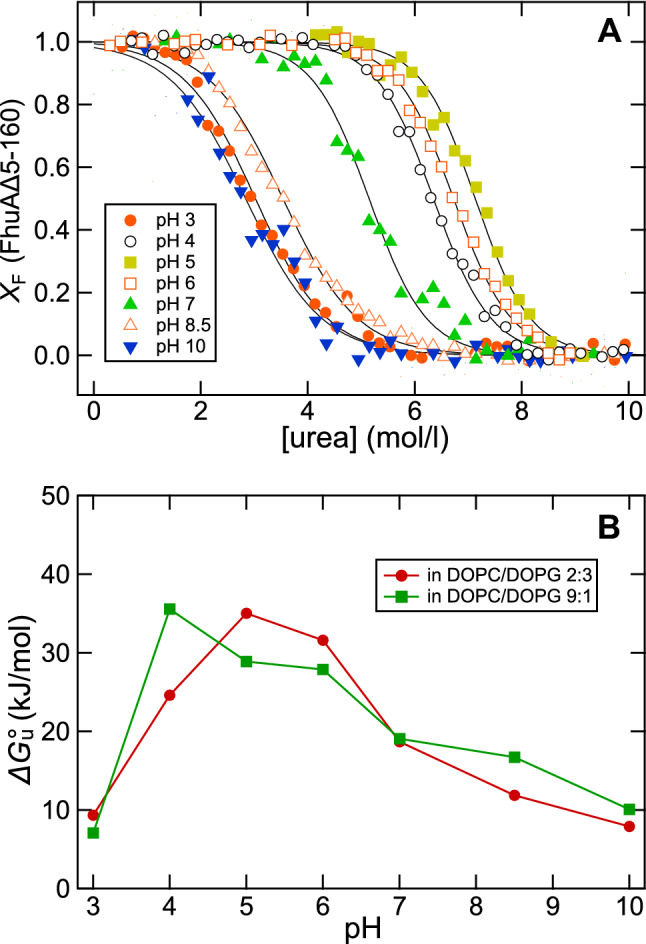
FhuAΔ5–160 is most stable near its isoelectric point. **A** Urea-induced unfolding of FhuAΔ5–160 at 40 °C in lipid bilayers (DOPC/DOPG 2:3) at pH 3 (red circle), pH 4 (black open circle), pH 5 (light green square), pH 6 (red square), pH 7 (green triangle), pH 8.5 (red tiangle), and pH 10 (blue downward pointing triangle), indicated stability is highest near pH 5. Fluorescence spectra similar to those shown in Fig. 2 were recorded. Intensity-weighted average fluorescence emission maxima and the fractions of folded FhuAΔ5-160 were calculated from the spectra using Eqs. 5 and 6. The fraction of folded FhuAΔ5–160 was plotted as a function of the urea concentration. The molar lipid/protein ratio was 100 at an FhuAΔ5–160 concentration of 0.64 μM. Equation (3) was fitted to the data to determine the effective free energies of folding $\Delta G^{\text{o}}_{{\text{U}} \rightarrow F} = -\Delta G^{\text{o}}_{{\text{F}} \rightarrow U}$. **B** The effective free energies of folding obtained from these fits to the data shown in panel A are shown as a function of pH (red circle). For comparisons, FhuAΔ5–160 was also reconstituted into lipid bilayers of DOPC and DOPG at a molar ratio of 9:1 and urea-induced unfolding was again monitored at the same pH values as described for panel A (green square)

The stability of FhuA in lipid bilayers also depends on pH and on the phospholipids present in a membrane (Fig. 8), e.g., negatively charged phospholipids may lead to a lower pH near the lipid bilayer water interface than in bulk solution. To examine at which pH FhuAΔ5-160 is most stable, we recorded its fluorescence spectra as a function of the urea concentration in a range from 0 to 10 M urea. Several unfolding experiments of FhuAΔ5-160 were performed, each at a different pH-value, ranging from 3 to 10. For these, FhuAΔ5-160 was reconstituted in mixed lipid bilayers composed of DOPC and DOPG containing either 10% or 60% of the negatively charged DOPG. From the recorded spectra, the $\left\langle {\lambda_{{\text{M}}} } \right\rangle$ were calculated and the fractions of folded FhuAΔ5-160 were determined for each dataset using Eq. 6. The urea-induced unfolding of FhuAΔ5-160 from bilayers of DOPC and DOPG (4:6) is shown in Fig. 8A, indicating that unfolding of FhuAΔ5-160 from bilayers of DOPC/DOPG (2:3) requires the highest concentrations of urea at pH 5, with a midpoint of unfolding at ~ 7.1 M urea. This is reflected in the free energies of unfolding (Fig. 8B) that are highest near the pI ~ 5.1 of FhuA, from pH 4 to 6, with $\Delta G{^\text{o}_{\rm u}}$ ≥ 20 kJ/mol. In contrast at the more extreme pH < 4 or pH > 9, the free energy of unfolding is less than half, with $\Delta G{^\text{o}_{\rm u}}$ ≤ 10 kJ/mol. The free energies of unfolding of FhuAΔ5-160 (Fig. 8B), calculated from the titrations were much lower when unfolding was performed at pH ≥ 7 or at highly acidic pH < 4, indicating that an increased net-charge of FhuA (calculated pI ~ 5.1) severely destabilizes it. At a pH sufficiently different from the pI, *i.e.,* at pH 3 or at pH ≥ 7, differences in the stabilities of FhuAΔ5-160 were $\Delta\Delta G{^\text{o}_{\rm u}}$ < 2.5 kJ/mol when reconstituted into bilayers of the two different DOPC/DOPG ratios. However, near its pI, the stability of FhuAΔ5-160 was most strongly affected by the content of negatively charged DOPG in the membrane, by $\Delta\Delta G{^\text{o}_{\rm u}}$ ~ 10 kJ/mol, depending on pH.

## Discussion

### Stability of FhuA in Detergent

In detergent micelles, FhuA has a lower thermostability than the smaller *β*-barrel OMPs as the midpoints of unfolding of wt-FhuA and FhuAΔ5-160 in thermal denaturation experiments were at lower temperatures than observed for the 16-stranded bacterial porins like OmpF (Sukumaran et al., 2004) or PorB class 2 protein in Zwittergent 3–14 (Minetti et al., 1997), for a review see, e.g., (Minetti & Remeta, 2006). Consistent with the present observations (Fig. 5, red circle), Bonhivers et al*.* (Bonhivers et al., 2001) reported that wt-FhuA (4.9 μM at pH 7.2 and at 1.3 mM LDAO) unfolds over a broad temperature range from ~ 61 to ~ 78 °C using differential scanning calorimetry (DSC). While a CD analysis in this previous study showed a single unfolding transition of the secondary structure of wt-FhuA with a midpoint at *T*_M_ ~ 72 °C, which is only slightly higher than *T*_M_ ~ 69 °C determined here by fluorescence spectroscopy (Fig. 5), the DSC scans indicated two changes of heat capacity, the change at the lower temperature contributing to ~ 47% of the total transition enthalpy. Bonhivers et al*.* concluded that this change in heat capacity was too large to reflect unfolding of the cork alone, but instead included unfolding of parts of the *β*-barrel domain.

For wt-FhuA, we found a much larger change in enthalpy upon unfolding with $\Delta H{_{\rm M}^\text{o}}$﻿ ~ 644 ± 28 kJ/mol than for FhuAΔ5-160 ($\Delta H{_{\rm M}^\text{o}}$﻿ ~ 255 ± 40 kJ/mol), with a difference of $\Delta\Delta H{_{\rm M}^\text{o}}$﻿ ~ 389 ± 68 kJ/mol (92 kcal/mol). In the previous DSC study on unfolding of wt-FhuA and of another mutant lacking most of the cork domain, FhuAΔ22-127, both solubilized in 1.3 mM LDAO at pH 7 (Bonhivers et al., 2001), a very similar difference of $\Delta\Delta H{_{\rm M}^\text{o}}$﻿ ~ 423 kJ/mol between enthalpies of unfolding was obtained.

Inspection of the crystal structures of FhuA (Ferguson et al., 1998b; Locher et al., 1998) indicated more than 60 hydrogen bonds between the *β*-barrel domain of FhuA and its N-terminal cork domain. A dense hydrogen-bonding network between the cork and the *β*-barrel and the additional unfolding of the 155-residue cork domain may explain the large difference in enthalpies of unfolding observed for wt-FhuA and FhuAΔ5-160 (FhuAΔ22-127). It would also explain why the cork domain greatly stabilizes the *β*-barrel domain against unfolding by either urea or by heat when FhuA is solubilized in LDAO micelles.

The observed $\Delta H{_{\rm M}^\text{o}}$ of unfolding of wt-FhuA or FhuAΔ5-160 are large in comparison to the $\Delta G{^\text{o}_{\rm u}}$ of either wt-FhuA or FhuAΔ5-160. There are no studies to date that experimentally determined both, $\Delta H{_{\rm M}^\text{o}}$﻿ and $\Delta G{^\text{o}_{\rm u}}$﻿, for a selected membrane protein in the same environment. However, similarly large ratios between $\Delta H{_{\rm M}^\text{o}}$ ﻿and $\Delta G{^\text{o}_{\rm u}}$﻿ as reported here, have been reported for many soluble proteins, *e.g.,* for lysozyme (Hawkes et al., 1984) or prion protein (Moulick & Udgaonkar, 2014). The reason for $\Delta H{_{\rm M}^\text{o}}$ >> $\Delta G{^\text{o}_{\rm u}}$ is that changes in $\Delta H{_{\rm M}^\text{o}}$ of proteins are usually compensated by changes of the entropy term $T \cdot \Delta S{_{\rm M}^\text{o}}$﻿ (Lumry & Rajender, 1970; Schellman et al., 1981). As known for soluble proteins (Schellman et al., 1981), a comparably small free energy of unfolding/folding leads to conformational flexibility necessary for protein function. The same principles apply to membrane proteins like FhuA as observed in the present study. A large enthalpy of unfolding is compensated by a gain of entropy. Present results indicate this applies to both domains, the *β*-barrel and the cork.

For another OMP, FepA, a $\Delta G{^\text{o}_{\rm u}}$﻿ of ~ 24.9 to 27.0 kJ/mol has been reported in 2% Triton-X-100 detergent at pH 7.2 and at ambient temperature (Klug et al., 1995). Under the selected experimental conditions, FepA is slightly more than half as stable as wt-FhuA in LDAO (Table 3) but still four to five times more stable than FhuAΔ5-160. FhuA and FepA are structurally very similar, both are 22-stranded *β*-barrels and both contain a cork domain of ~ 155 residues that is connected to a *β*-barrel domain by a dense network of hydrogen bonds (Buchanan et al., 1999; Ferguson et al., 1998b; Locher et al., 1998). The differences in the stabilities of the two iron-siderophore receptors may be caused by the different chemical structures of the detergents used for solubilization. A more likely cause is differences in the accessible surface area (ASA). The loss of ASA of FepA upon association of the cork domain inside the *β*-barrel domain has been reported to 2,798 Å^2^ (Buchanan et al., 1999) while for FhuA, this loss was described to be more than 5000 Å (Ferguson et al., 1998b). The greater reduction of ASA by ~ 2202 Å^2^ could explain why wt-FhuA was more stable than FepA, as heat capacities, enthalpies, and entropies depend on the solvent-ASA of the protein (Haltia & Freire, 1995; Privalov & Gill, 1988). The observed $\Delta G{^\text{o}_{\rm u}}$﻿ ~ 45 kJ/mol of wt-FhuA (Table 1) in LDAO micelles (10 mM HEPES, pH 7.0) was lower than observed for the 8-stranded *β*-barrel OmpA, for which a $\Delta G{^\text{o}_{\rm u}}$ of ~ 60 kJ/mol was reported in LDAO micelles in Borate buffer pH 10 (Pocanschi et al., 2013), while $\Delta G{^\text{o}_{\rm u}}$﻿ was ~ 65 ± 2 kJ/mol when solubilized in octylmaltoside in glycine buffer at pH 10 (Andersen et al., 2012). OmpA (theoretical pI ~ 5.5) is highly negatively charged at pH 10 and in view of the pH dependence of $\Delta G{^\text{o}_{\rm u}}$﻿ of FhuA observed in this study (Fig. 8), OmpA could be even more stable at neutral pH or closer to the pI. The interior of the *β*-barrel domain of OmpA has been described to contain an extended hydrogen-bonding network and eight cavities with a total solvent-ASA of 376 Å^2^ (Pautsch & Schulz, 2000). As OmpA is more compact with relatively far fewer surface exposed residues than FhuA, the more limited solvent accessibility of the entire polypeptide chain may also explain the high activation energy observed for unfolding of OmpA from large unilamellar vesicles (Pocanschi et al., 2006) and the long incubation times that were required for its unfolding from LDAO micelles or amphipathic polymers (Pocanschi et al., 2013).

### Stability of wt-FhuA and FhuAΔ5-160 in Lipid Bilayers

Analysis of unfolding of FhuA indicated $\Delta G{^\text{o}_{\rm u}}$﻿ of FhuAΔ5-160 in bilayers was larger than observed for the LDAO-micelle-solubilized form, with a gain of stability of $\Delta\Delta G{^\text{o}_{\rm u}}$ ﻿(FhuAΔ51-60) ~ 15 ± 8 kJ/mol, while the gain of stability of wt-FhuA was $\Delta\Delta G{^\text{o}_{\rm u}}$﻿ (wt-FhuA) ~ 38 ± 16 kJ/mol. These calculated $\Delta\Delta G{^\text{o}_{\rm u}}$ for bilayer-inserted vs. LDAO-solubilized forms of FhuA were modest in comparison to the strong shifts of the midpoints of the temperature-induced unfolding observed after reconstitution of FhuA in lipid bilayers. This suggests that much of the additional heat is used to compensate for entropy effects. From the dependence of the urea-induced unfolding of FhuA from bilayers on temperature (Fig. 7 and Table 3), enthalpies of unfolding may be estimated to $\Delta H{^\text{o}_{\rm u}}$﻿ ~ 1000 kJ/mol for wt-FhuA (both domains) and to $\Delta H{^\text{o}_{\rm u}}$﻿ ~ 350 kJ/mol for FhuAΔ5-160. These differences in $\Delta H{^\text{o}_{\rm u}}$﻿ are ~ 40 to 50% larger than observed for unfolding of FhuA from LDAO micelles (Table 2) and support the conclusion that the stabilization of FhuA by the lipid bilayer has a strong contribution from the entropy term $T\cdot\Delta S{_{\rm M}^\text{o}}$﻿, as $\Delta G{^\text{o}_{\rm u}}$ is much smaller than $\Delta H{^\text{o}_{\rm u}}$.

When FhuA unfolds, the conformational entropy of FhuA increases while increased unfavorable contacts between hydrophobic and polar groups or solvent molecules result in negative contributions to $\Delta S{_{\rm M}^\text{o}}$. For unfolding of hen lysozyme, a soluble protein, the total change in entropy of unfolding, $\Delta S{_{\rm M}^\text{o}}$, has been described as the sum of negative entropy changes by water-exposure of non-polar surface, $\Delta S{^\text{o}_{\rm hyd}}$, and positive entropy contributions $\Delta S{^\text{o}_{\rm res}}$﻿, dominated by unfolding of the polypeptide chain (Baldwin, 1986; Baldwin & Muller, 1992). Contrary to the comparably large and constant $\Delta S{^\text{o}_{\rm res}}$, the negative $\Delta S{^\text{o}_{\rm hyd}}$ of lysozyme unfolding increased with temperature (the absolute value decreased), which was necessary for unfolding of lysozyme (Baldwin, 1986). For membrane proteins, the ‘solvent’ is more complex as membrane proteins are exposed to both hydrophobic and polar environments. While the core of the lipid bilayer is hydrophobic, some surface of the membrane protein faces the aqueous environment on both sides of the membrane. For FhuA, the polar surface also includes some of the inner surface of the β-barrel that is not in contact with the cork domain. Since the stabilization of FhuA caused by the lipid bilayer is apparently dominated by entropy and because the difference in conformational entropy $\Delta S{^\text{o}_{\rm res}}$﻿ of FhuA that is bilayer inserted vs. $\Delta S{^\text{o}_{\rm res}}$ of LDAO-solubilized FhuA are small, the difference in stability of FhuA in these two environments would be caused by an unfavorable $\Delta S{^\text{o}_{\rm hyd}}$, caused by unfavorable solvent interactions of the LDAO-micelle-solubilized FhuA. Conversely, a favorable $\Delta S{^\text{o}_{\rm hyd}}$﻿ may be the driving force for FhuA insertion into lipid bilayers. Large contributions of solvent entropy to folding of soluble protein were recently reported for a soluble protein (Heinz & Grubmüller, 2021).

Plançon et al. (Plançon et al., 2002) showed that formation of complexes at 1:1 stoichiometry of wt-FhuA with the Phage T5 protein pb5 at pH 7.0 greatly stabilizes wt-FhuA and pb5 against thermal denaturation. In DSC experiments at ~ 5 μM FhuA, complexes at ~ 200 dodecylmatoside/FhuA showed a single unfolding transition at *T*_M_ ~ 86 °C, indicating that pb5 stabilizes FhuA to an extent (Δ*T*_Μ_ >  ~ 14 °C) that is very similar to the stabilization observed here for wt-FhuA in lipid bilayers, which did not completely denature below 90 °C (Fig. 5, red triangle). Both integration into lipid bilayers or binding of the large pb5 (68 kDa) shield FhuA from unfavorable interactions with solvent, which supports our conclusion that destabilization of FhuA in detergent micelles is caused by entropically unfavorable interactions of folded FhuA with the solvent.

**Fig. 9:**
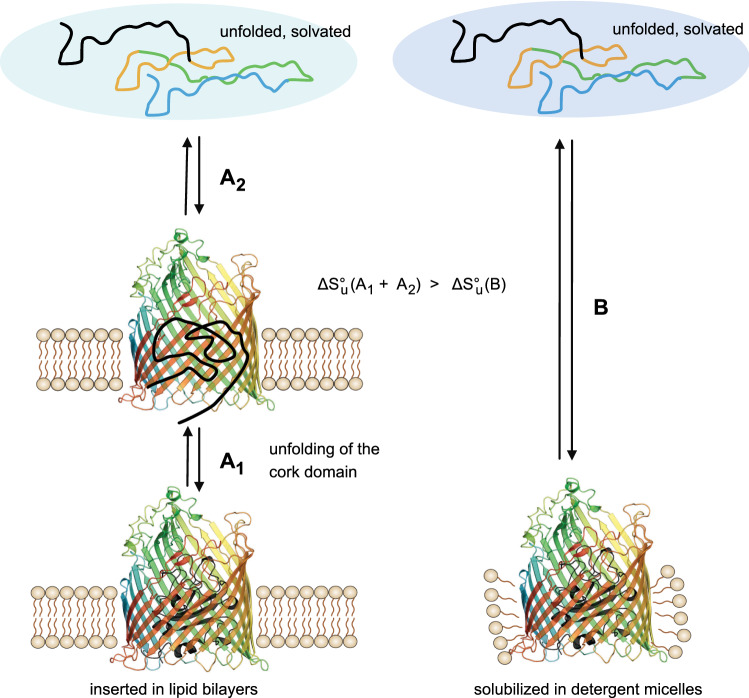
Lipid bilayers stabilize the *β*-barrel domain of wt-FhuA and facilitate the independent unfolding of its cork domain in a three-state unfolding process. In contrast, solubilization of wt-FhuA in LDAO micelles leads to a two-state unfolding process of wt-FhuA, in which unfolding of both domains is coupled to another. In comparison to solubilization in LDAO micelles, the insertion of FhuA into the lipid bilayer is characterized by a higher gain of solvation entropy

The stabilization of the *β*-barrel domain by the lipid bilayer was sufficient to allow for an independent unfolding of the cork domain from bilayers of DOPC and DOPG (2:3) (Figs. 7 and 9). Unfolding of the *β*-barrel domain of wt-FhuA with a covalently linked unfolded cork domain is not the very same process as unfolding of FhuAΔ5-160, in which the cork domain is completely absent. Given the large amount of 60 hydrogen bonds between the cork domain and the *β*-barrel domain, it is possible that some hydrogen bonds remain intact upon unfolding of the cork domain, leading to some residual stabilization of the β-barrel domain, which appears to be slightly more stable in wt-FhuA than in FhuAΔ5-160 (Fig. 7). The OM of *E. coli* contains LPS in the outer leaflet and a binding site for LPS has been identified for FhuA by X-rays crystal structure analysis (Ferguson et al., 2000). It is very likely that in the native *E. coli* membrane, the stabilization of the *β*-barrel domain of FhuA in native membranes is even greater than observed here for FhuA in model membranes.

$\Delta G{^\text{o}_{\rm u}}$ of OM proteins from lipid bilayers have been determined previously mostly at highly basic pH > 9 (Chaturvedi & Mahalakshmi, 2018; Hong et al., 2013; Iyer et al., 2018; Pocanschi et al., 2006, 2013; Sanchez et al., 2008) or very acidic pH < 4 (Moon et al., 2011; Moon et al., 2013) for smaller 8 or 12 stranded *β*-barrel membrane proteins like OmpA or OmPlA. As the OMPs from *E. coli* examined to date have in common that their isoelectric points are all in the range between pI ~ 5.0 and pI ~ 5.9 (Kleinschmidt, 2006, 2015), in most studies on their folding and stability, they are typically either negatively charged at pH > 9 or positively charged at pH < 4. This may lead to destabilizing repulsions between equally charged side chains but is necessary to avoid aggregation and, therefore, reduced folding yields as reported for folding of OmpA at pH 7 (Surrey & Jähnig, 1995). When integrated into lipid bilayers of LUVs, the small *β*-barrel OmpA has a high activation energy of unfolding (Pocanschi et al., 2006). Reasons may be that with only 8 or 12 *β*-strands, the smaller *β*-barrels like OmpA are relatively compact, leading to a lower probability of local destabilizations of the *β*-barrel than for the larger *β*-barrels. In denaturant induced unfolding of FhuA, limited accessibility of the smaller lumen of *β*-barrels like OmpA to solvent or denaturant could be another reason for increased resistance against unfolding.

## Abbreviations

CD: Circular dichroism
DOPC: 1,2-Dioleoyl-*sn*-glycero-3-phosphocholine
DOPE: 1,2-Dioleoyl-*sn*-glycero-3-phosphoethanolamine
DOPG: 1,2-Dioleoyl-*sn*-glycero-3-phosphoglycerol
DSC: Differential scanning calorimetry
EDTA: Ethylenediaminetetraacetic acid
FepA: Ferric enterobactin receptor
FhuA: Ferric hydroxamate uptake protein A
FhuAΔ5-160: β-Barrel domain of FhuA
FPLC: Fast protein liquid chromatography
HEPES: (4-(2-Hydroxyethyl)-1-piperazineethanesulfonic acid)
LDAO: *N*-Lauryl-*N*, *N*-dimethylammonium-*N*-oxide
Mcps: Million counts per second
Mdeg: Millidegrees
OM: Outer membrane
OmpA: Outer membrane protein A
OMP: Outer membrane protein
PAGE: Polyacrylamide gel electrophoresis
SDS: Sodium dodecylsulfate
SUVs: Small unilamellar vesicles, TMP, transmembrane protein, Tris, tris(hydroxymethyl)aminomethane
Triton-X-100: 4-(1,1,3,3-Tetramethylbutyl)-phenyl-polyethylenglykol
wt: Wild-type
